# Cost-effectiveness of social media advertising as a recruitment tool: A systematic review and meta-analysis

**DOI:** 10.1017/cts.2023.596

**Published:** 2023-08-07

**Authors:** Vladislav Tsaltskan, Roel Sanchez Baez, Gary S. Firestein

**Affiliations:** Department of Medicine, University of California San Diego, La Jolla, CA, USA

**Keywords:** Social media, cost, recruitment, meta-analysis, systematic review

## Abstract

**Background:**

Recruitment of study participants is challenging and can incur significant costs. Social media advertising is a promising method for recruiting clinical studies and may improve cost efficiency by targeting populations likely to match a study’s qualifications. Prior systematic reviews of social media as a recruitment tool have been favourable, however, there are no meta-analyses of its cost-effectiveness.

**Methods:**

Studies evaluating recruitment costs through social media and non-social media methods were identified on MEDLINE and EMBASE. Articles were screened through a two-step process in accordance with PRISMA guidelines. Cost data were extracted from selected articles and meta-analyzed using the Mantel-Haenszel method. The primary outcome was the relative cost-effectiveness of social media compared to non-social media recruitment, defined as the odds ratio of recruiting a participant per US dollar spent. The secondary outcome was the cost-effectiveness of social media recruitment compared to other online recruitment methods only.

**Results:**

In total, 23 studies were included in the meta-analysis. The odds ratio of recruiting a participant through social media advertising compared to non-social media methods per dollar spent was 1.97 [95% CI 1.24–3.00, *P* = 0.004]. The odds ratio of recruiting a participant through social media compared to other online methods only was 1.66 [95% CI 1.02–2.72, *P* = 0.04].

**Conclusions:**

Social media advertising may be more cost-effective than other methods of recruitment, however, the magnitude of cost-effectiveness is highly variable between studies. There are limited data on newer social media platforms and on difficult-to-reach populations such as non-English speakers or older individuals.

## Background

Effective recruitment of research study participants is a major challenge in clinical research. Up to 50% of studies close early due to inadequate participant accrual [1,2], with non-interventional studies typically faring worse [3]. In fact, many study sites fail to enrol a single participant [4]. Rare diseases and underserved or underrepresented populations such as non-English speakers tend to be more difficult to recruit [5–7]. Traditional recruitment approaches for clinical studies include in-person identification of participants from physician offices or health fairs, printed flyers and posters or advertisements on radio, television or more recently on the Web [8]. However, these approaches have several disadvantages. They are often slow and inefficient, require large amounts of investigator time commitment and can be very expensive relative to the number of recruited participants [8].

Investigators increasingly use social media to recruit individuals for clinical research studies [9–11]. Social media recruitment methods can be divided into two broad categories. These include unpaid methods, typically involving researchers posting information on their study using their personal or institutional social media account, and paid methods, which use advertisements placed through each social media platform’s dedicated ad service [12]. Depending on the platform, these ads can be targeted to specific users in various ways. Researchers can use these ad targeting techniques to identify users more likely to qualify for or participate in their study. Additionally, once users click on an ad, deidentified data about them can be collected. This can inform investigators about advertising reach and target audience demographics. However, there are ethical issues around privacy and data safety in using social media advertising for these purposes, and care must be taken to avoid accidental reidentification of participants.

The effectiveness of social media recruitment is highly variable and depends on the specific study population [11]. Social media advertising costs are widely divergent, and advertisement clickthrough rates can vary between studies and are hard to predict in advance. Cost-effectiveness of this modality, therefore, has been difficult to study systematically. Previous systematic reviews identified generally favourable costs, with social media ranging from $0 to $517 per participant, compared to $19–$777 for traditional methods [11,13]. However, there are no published meta-analyses of the cost-effectiveness of social media recruitment compared to non-social media methods. We now present a systematic review and meta-analysis of the cost-effectiveness of paid social media advertising for recruiting participants to clinical studies.

## Methods

### Study Inclusion and Exclusion Criteria

Any study directly comparing the cost of paid social media advertising versus non-social media recruitment methods to a clinical study was considered for inclusion. Social media platforms included were Facebook, Instagram, Twitter, Reddit, Linkedin, Snapchat, Youtube and TikTok. Non-social media methods could be any other recruiting method with a reported cost. Studies could include personnel time, development costs, recurring advertisement fees, supply costs and other miscellaneous expenses as part of the total cost; however, this had to be reported consistently across different recruitment methods. Included studies were expected to be secondary analyses of existing data, and allowable designs of the primary studies were randomised controlled trials, longitudinal studies, cohort studies or cross-sectional studies. Due to continuing changes in social media advertising algorithms, only studies within the last ten years were included. Single-arm studies of social media without a comparator, studies comparing only different social media platforms or studies of social media for purposes other than recruitment were excluded. Studies that only used no-cost social media or non-social media recruitment methods or did not report cost data were also excluded. Additionally, studies that recruited less than ten participants through social media or non-social media methods were excluded.

### Outcomes

The primary outcome measure was the relative cost-effectiveness of social media recruitment compared to non-social media recruitment. This was calculated as the odds ratio of enrolling a participant into a study per dollar spent on each recruiting method. This approach was selected in order to minimise the effects of inter-study variability. By directly comparing costs of recruitment methods within each individual study, we could control for confounding effects such as differences in population, cost calculation, language and study type.

The secondary outcome measure was the relative cost-effectiveness of social media advertising compared to other forms of paid online advertisements including non-social media-based web ads (such as on Google) and mobile app ads. This was calculated as an odds ratio using the same method as the primary outcome. A study needed to enrol at least ten participants using this approach in order to qualify for inclusion in the secondary analysis.

### Search Strategy

The search strategy for the meta-analysis followed the Preferred Reporting Items for Systematic Reviews and Meta-Analyses guidelines [14]. MEDLINE and EMBASE were searched over a three-month period ending in November 2022 to identify candidate studies. A detailed search strategy is presented in Supplemental Figure 1.

### Article Screening

After completion of the MEDLINE and EMBASE search, identified articles were screened using a two-step process. The initial step consisted of abstract screening only, in order to eliminate irrelevant papers or review articles. The second step involved whole-article screening, and papers were included or excluded at this step based on the above criteria. Two authors (VT and RS) independently reviewed candidate articles for inclusion. Included articles were assessed for quality using the Joanna Briggs Institute (JBI) checklist for Quasi-Experimental Studies [15].

### Data Extraction

Relevant data were manually extracted from each included article including the study location and type, recruited population, social media platforms used, other forms of recruitment used, total number of participants recruited by each method and total cost of each recruitment method. For the purposes of this study, Web-based recruitment that was not through social media (such as Google ads) was pooled with other non-social media methods. Any recruitment methods that did not report an associated cost, or reported a cost of zero, were excluded from the analysis.

### Cost Normalisation

Any costs or prices reported in currencies other than US dollars were first converted to US dollars using the average annual currency conversion rate during the year of study publication. All costs were then adjusted for inflation to November 2022 US dollars. Cost comparisons between studies were reported as medians and interquartile ranges (IQR).

### Statistical Analysis

Individual odds ratios were calculated for each study and outcome as described in the outcomes section above. Variances for each odds ratio were then obtained and meta-analyzed using the Mantel-Haenszel method [16]. Due to a high degree of heterogeneity expected in the results, the random effect model was selected. A formal heterogeneity analysis was subsequently performed in order to confirm that the random effect model was appropriate. Results were reported as an odds ratio and 95% confidence interval (CI). All analysis was performed using Revman 5.4.1 (The Cochrane Collaboration, 2020) and R Statistical Software (R Core Team, 2022).

## Results

### Article Screening and Risk of Bias Assessment

**Figure 1.**
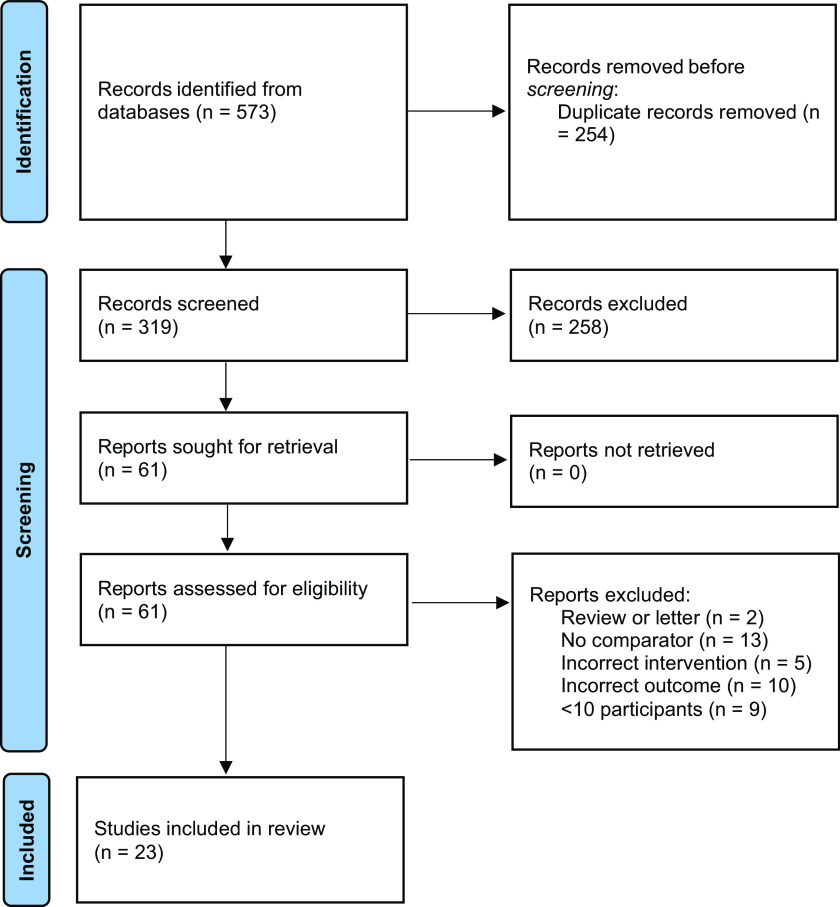
Study inclusion flow diagram.

A total of 319 unique articles were identified during the search process. Of these, 258 were excluded during the abstract screening process, leaving 61 for full-text review. In total, 38 articles were subsequently excluded during the full-text review: 13 were excluded due to lacking a non-social media comparator, 10 because they did not report costs, five because they did not use social media for recruitment, nine had less than 10 participants in either the social media or non-social media group and two were reviews. Twenty-three remaining articles were included in the analysis [17–39]. Because all included studies were secondary analyses, a risk of bias assessment could not be performed directly. Instead, all 23 identified studies were appraised using the JBI checklist for Quasi-Experimental Studies and were found to be appropriate for inclusion in the meta-analysis. A complete inclusion flow diagram is presented in Fig. 1.

### Characteristics of Included Studies

All 23 studies included in the meta-analysis were secondary analyses of previously collected data. In 13 of these, the primary study was a randomised controlled trial. The primary study was a cohort study in seven articles and a cross-sectional study in three articles. The most commonly studied disease, with seven included articles, was substance use including alcohol and tobacco [22–24,26,29,32,35]. Six articles evaluated reproductive and sexual health including sexually transmitted infections, contraceptive usage, and prenatal counselling [17,19,27,32,33,39]. The remaining ten articles evaluated various diseases such as diabetes mellitus [34], autism spectrum disorder [18] and prostate cancer [36]. Only two studies involved a pharmacological intervention [27,33], with most of the remaining studies evaluating behavioural interventions or surveys. Fourteen studies were conducted in the United States, five in Australia, three in Canada and one in New Zealand. Target populations were highly variable, although many studies specifically aimed to recruit younger individuals [19,31–35,39].

All of the studies used Facebook as a recruiting tool. Two studies also evaluated Instagram [19,32] and two also studied Twitter [22,31]. Youtube, Reddit, Linkedin and Snapchat were each evaluated by one study [31,32,37]. Included studies used between one and seven non-social media recruitment methods. Non-social media methods that were evaluated included in-person methods such as physician’s offices or health fairs, mailed advertisements, posters, newspaper ads, TV ads, radio ads and non-social media online advertisements such as email, Web ads and mobile apps. Table 1 contains a description of included study characteristics.

**Table 1.** Characteristics of included studies

Author	Year	Country	Population	Study type	Social media used	Other methods used	Total cost ($USD)	Number of participants	JBI checklist
Adam	2016	Canada	Pregnant women	RCT	FB	Posters, Newspapers, TV, In-person	$1,457	70	Include
Ahmed	2020	USA	Individuals with autism	Cohort	FB	Radio	$17,077	487	Include
Barney	2021	USA	Girls 15–19 using LARCs	Cohort	FB, IG	In-person	$131,490	636	Include
Batterham	2014	Australia	Adults	Cross Sectional	FB	Mail	$49,222	4869	Include
Benham	2021	Canada	Women with PCOS	RCT	FB	Posters	$4,628	46	Include
Byaruhanga	2019	Australia	Smokers	RCT	FB, Twitter	Web, Email, Newspaper, Radio, Magazine, Posters, Phone	$36,729	635	Include
Carlini	2015	USA	Brazilian Americans	Cross Sectional	FB	Web, Email, Newsletter	$10,687	690	Include
Carter-Harris	2016	USA	Smokers	Cross Sectional	FB	Newspaper	$2,121	361	Include
Dobkin	2020	USA	Adults with PD	Cohort	FB	Web	$89,846	1838	Include
Faro	2021	USA	Smokers	Cohort	FB	Web, In-person	$54,074	1487	Include
Guthrie	2019	USA	Postmenopausal women	RCT	FB	Mail	$131,668	302	Include
Kayrouz	2016	Australia	Arabic speakers	Cohort	FB	Radio, Newspaper, Email	$3,727	81	Include
Medina-Ramirez	2020	USA	Spanish-speaking smokers	RCT	FB	Web, TV, posters	$154,170	1320	Include
Morgan	2013	Australia	Adults at risk for depression	RCT	FB	Web	$10,765	790	Include
Musiat	2016	Australia	Adults 18–25	RCT	FB, Twitter, Youtube	Web, Recruitment agency	$9,066	84	Include
Parker	2021	USA	GSM age 15–29	RCT	FB, IG, Reddit, Snapchat	Web, Apps, In-person	$64,426	174	Include
Rait	2015	USA	Adolescents 13–17	Cohort	FB	Posters, In-person	$16,901	184	Include
Raviotta	2016	USA	Men 18–25	RCT	FB	Posters, Newspaper, Email, In-person	$14,241	155	Include
Rosser	2022	USA	GSM with prostate cancer	RCT	FB	Newspapers, Posters, Mail, Apps, Web	$21,727	401	Include
Salvy	2020	USA	Diabetics 18–30	RCT	FB	In-person, Mail	$26,556	79	Include
Volkova	2017	New Zealand	Adults	RCT	FB, Linkedin	Web, Radio, Email, Magazine, Posters, In-person	$7,155	1199	Include
Wasfi	2021	Canada	Adults	Cohort	FB	Mail, Newspapers, TV, Web, In-person	$34,609	1791	Include
Woods	2021	USA	Age 16–25 with STI	RCT	FB	In-person	$25,786	21	Include

FB = Facebook; GSM = gender and sexual minorities; IG = Instagram; LARCs = long-acting reversible contraceptives; PCOS = polycystic ovarian syndrome; PD = Parkinson’s Disease; RCT = randomised controlled trial; STI = sexually transmitted infection.

### Participant Recruitment and Cost-Effectiveness

Included studies recruited between 21 and 4869 participants and spent between USD $1457 and $154,170 on recruitment. The median cost per enrolled participant through social media was $45.51 [IQR $13.92–$134.81] and the median cost per enrolled participant through other methods was $74.89 [IQR $6.57–$187.15], however, this trend was not statistically significant (*P* = 0.14).

**Figure 2.**
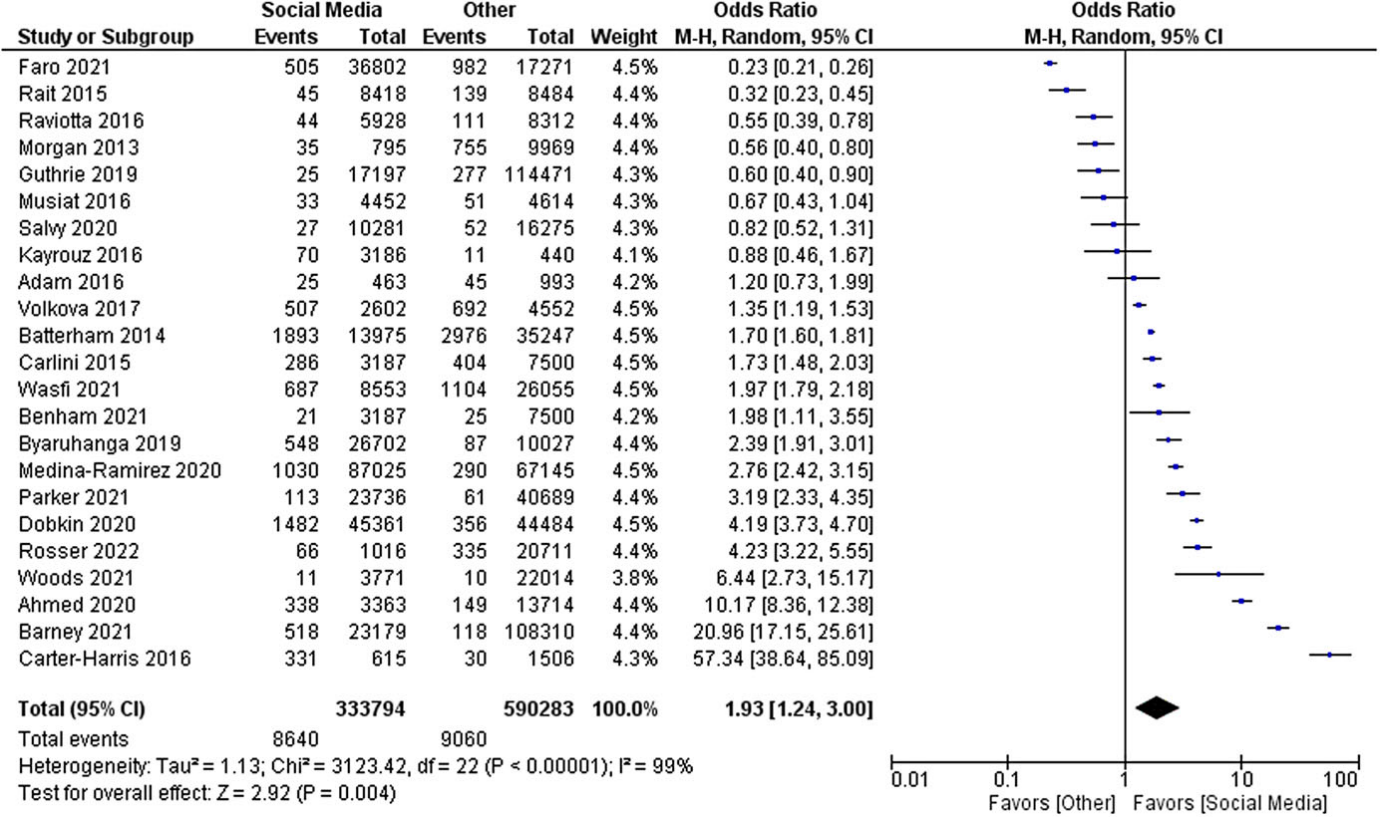
Forest plot of primary outcome.

**Figure 3.**
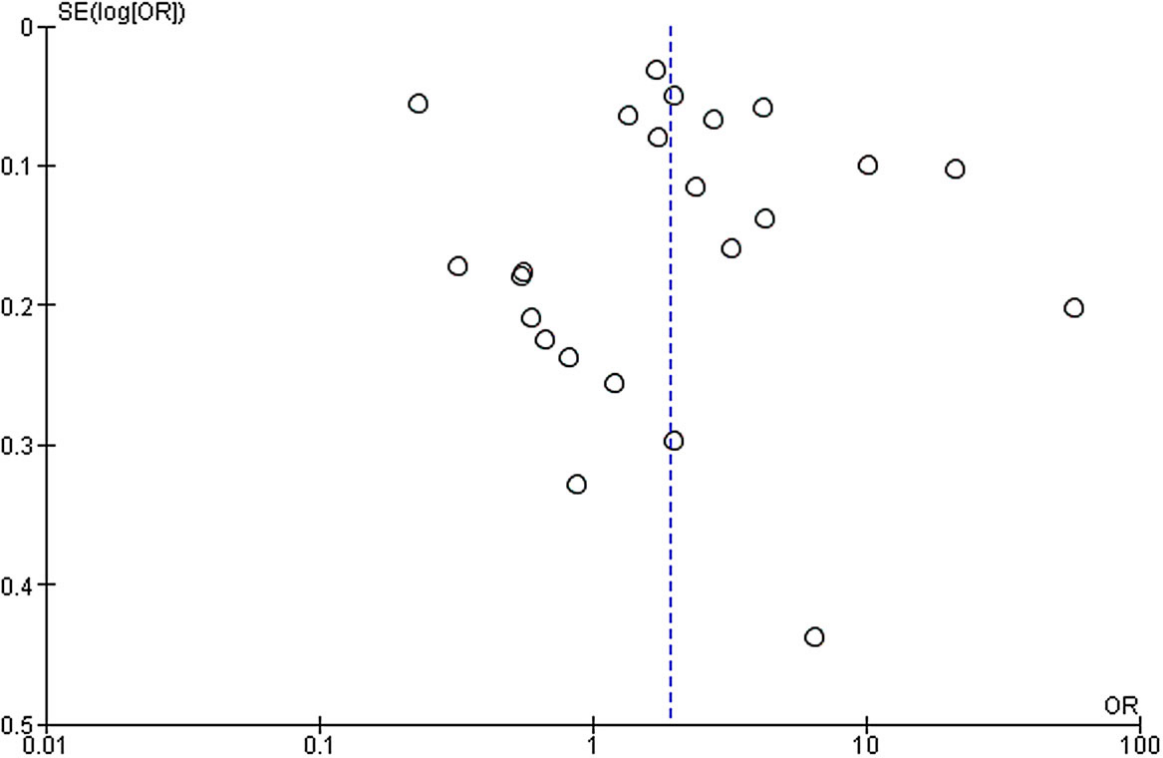
Funnel plot of primary outcome.

The primary outcome of relative cost-effectiveness, defined as the odds ratio of recruiting a participant per dollar spent on social media compared to other methods, was 1.93 [95% CI 1.24–3.00, *P* = 0.004]. Therefore, for a fixed amount of spending on recruitment methods, an investigator could expect to enrol nearly twice as many participants using social media compared with non-social media methods. Heterogeneity analysis was highly significant for the presence of heterogeneity in the sample, as expected (*I* [2] = 99%, *P* < 0.001). The forest plot for this outcome measure is displayed in Fig. 2. A funnel plot was also generated to evaluate for possible publication bias. No publication bias was evident as shown in Fig. 3.

**Figure 4.**
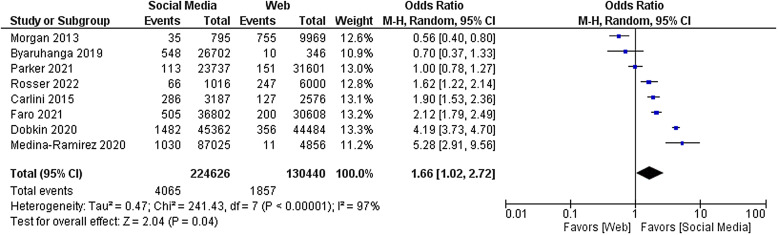
Forest plot of secondary outcome.

Of the 23 studies included in the primary analysis, eight studies enrolled at least ten participants using paid online methods other than social media and were eligible for inclusion in the secondary analysis [22,23,25,26,29,30,32,36]. The odds ratio of recruiting a participant per dollar spent on social media advertising compared to other online methods was 1.66 [95% CI 1.02–2.72, *P* = 0.04]. As in the primary analysis, there was a high amount of heterogeneity in this sample (*I* [2] = 97%, *P* < 0.001). The forest plot for this analysis is displayed in Fig. 4.

## Discussion

In this meta-analysis, we evaluated the cost-effectiveness of social media as a participant recruitment tool for clinical studies. This is, to our knowledge, the first systematic cost evaluation of social media recruitment in clinical studies. We found that social media was about twice as cost-effective as non-social media methods overall for recruiting participants, although the degree of cost-effectiveness was extremely variable between studies. When we restricted our analysis to comparing social media to other forms of paid online advertising, we again found that social media had superior cost-effectiveness, although the odds ratio was lower than in the primary analysis. Because cost-effectiveness was compared directly between methods within each study, we avoided some confounding factors such as expected recruitment numbers and geographic or temporal factors affecting recruitment. By including a relatively large number of studies, we were able to mitigate the wide variability of the underlying studies.

Despite this mitigation, high variability remained a major limitation in our analysis. This variability existed in the patient population, study type, location and recruitment methods, resulting in a wide difference in effect sizes even in studies with similar standard errors, as can be seen in Fig. 3. Additionally, the methods of determination of costs of non-social media recruitment varied considerably, with some but not all studies including the cost of employee time. Due to this degree of variability, we could not control for confounding factors in the primary analysis. Thus, even though the overall effect favoured social media, it would be difficult for an investigator to predict the cost-effectiveness of social media in their particular study before implementing the recruitment campaign.

While all included studies evaluated Facebook as a recruitment tool, other social media platforms were not studied frequently. Only one study evaluated Youtube for recruitment [31], even though Youtube is the second largest social media platform after Facebook [40], and no data are available on newer social networks such as TikTok or Nextdoor. Advertising algorithms within social media are constantly being updated, so data from older studies may no longer be accurate.

Only two studies [28,29] focused on non-English speakers; one noted significant benefit from a social media campaign, and the other found no difference. It is therefore impossible to extrapolate from these data if the benefit of social media recruitment extends to non-English speaking individuals. Although many studies focused on younger participants, only two studies focused on older individuals [25,36], who are increasingly active on social media [41]. Therefore, more studies are needed to evaluate the effectiveness of social media recruitment in these groups.

In summary, social media advertising is a powerful recruitment tool for clinical investigators and is generally more cost effective than traditional recruitment methods. However, the benefits vary widely depending on the study and population. Additional studies are needed to evaluate less commonly used social media platforms and hard-to-reach individuals and rigorously control for confounders that could affect the results.

## Supporting information

Tsaltskan et al. supplementary material Tsaltskan et al. supplementary material

## Supplementary material

The supplementary material for this article can be found at https://doi.org/10.1017/cts.2023.596.
