# Supplementary material for: Cost-effectiveness of social media advertising as a recruitment tool: A systematic review and meta-analysis
Source: J Clin Transl Sci. 2023 Aug 7;7(1):e180. doi: 10.1017/cts.2023.596 (PMC10514690; doi:10.1017/cts.2023.596)
Supplement: Tsaltskan et al. supplementary material [file S2059866123005964sup001.docx]

**Supplemental Figure 1: Search strategies**

**MEDLINE Mesh terms:**

(("social media"[MeSH Terms] OR ("social"[All Fields] AND "media"[All Fields]) OR "social media"[All Fields])

AND

("recruit"[All Fields] OR "recruited"[All Fields] OR "recruiter"[All Fields] OR "recruiters"[All Fields] OR "recruiting"[All Fields] OR "recruitment"[All Fields] OR "recruitments"[All Fields] OR "recruits"[All Fields]))

AND

((y_10[Filter]) AND (clinicaltrial[Filter] OR randomizedcontrolledtrial[Filter]))

**EMBASE search terms:**

'social media recruitment cost'

OR

(('social'/exp OR social) AND ('media'/exp OR media) AND ('recruitment'/exp OR recruitment) AND ('cost'/exp OR cost))

AND

[2012-2022]/py
